# Molecular Mechanism for Stress-Induced Depression Assessed by Sequencing miRNA and mRNA in Medial Prefrontal Cortex

**DOI:** 10.1371/journal.pone.0159093

**Published:** 2016-07-18

**Authors:** Ke Ma, Li Guo, Aiping Xu, Shan Cui, Jin-Hui Wang

**Affiliations:** 1 Qingdao University, School of Pharmacy, Shandong, China; 2 State Key Lab of Brain and Cognitive Science, Institute of Biophysics, Chinese Academy of Sciences, Beijing, China; 3 University of Chinese Academy of Sciences, Beijing, China; 4 College of Life Science, University of Science and Technology of China, Hefei, Anhui, China; Chiba University Center for Forensic Mental Health, JAPAN

## Abstract

**Background:**

Major depression is a prevalent mood disorder. Chronic stress is presumably main etiology that leads to the neuron and synapse atrophies in the limbic system. However, the intermediate molecules from stresses to neuronal atrophy remain elusive, which we have studied in the medial prefrontal cortices from depression mice.

**Methods and Results:**

The mice were treated by the chronic unpredictable mild stress (CUMS) until they expressed depression-like behaviors confirmed by the tests of sucrose preference, forced swimming and Y-maze. High-throughput sequencings of microRNA and mRNA in the medial prefrontal cortices were performed in CUMS-induced depression mice versus control mice to demonstrate the molecular profiles of major depression. In the medial prefrontal cortices of depression-like mice, the levels of mRNAs that translated the proteins for the GABAergic synapses, dopaminergic synapses, myelination, synaptic vesicle cycle and neuronal growth were downregulated. miRNAs of regulating these mRNAs are upregulated.

**Conclusion:**

The deteriorations of GABAergic and dopaminergic synapses as well as axonal growth are associated with CUMS-induced depression.

## Introduction

Major depressive disorder is featured as anhedonia and low self-esteem. The chronic stress to the genetically susceptible subjects leads to the deficits of monoamine, brain-derived neurotrophic factor and hypothalamus-pituitary-adrenal pathway [1–6], which induce neuronal atrophy in brain reward circuits, such as prefrontal cortex and nucleus accumbens, in depression patients and depression-like animals [7–13]. Genetic analyses by blood and dermal cells indicate that the deficits of numerous genes are related to genetic susceptibility for major depression [14–18]. However, these data may not reflect how stress environments influence these molecules in the brain that mediate pathological changes in major depression, such that gene analyses in depression-related brain regions are needed [19–22].

The analyses of gene expression in the limbic system from depression subjects indicate that many genes are involved in major depression, in which some of them differ from the analyses in the peripherals. The changes of these genes may lead to the depressed cell metabolisms and synapse functions [19–23]. The change of gene expression in the brain is presumably caused by a process that environmental factors make epigenetic dysregulation and in turn lead to major depression [24–26]. This assumption is granted by analyzing microRNA (miRNA) in certain brain areas from the postmortem tissues of major depression patients [27–31] and from the brain tissues of depression-like mice [32, 33]. The reaction chain from stress to miRNA dysregulation, messenger RNA (mRNA)/protein expression alternations and neuronal atrophy is presumably related to major depressive disorders. However, the molecules identified for major depression appear varied in these reports. The inconsistence may be due to the analyses applied in these studies from the different experiment models, brain areas, animal species and/or the approaches by analyzing mRNA or miRNA. For instance, the changes of miRNAs in the reports without the alternations of their targeted mRNAs in other reports imply that these miRNAs may not reach the thresholds to regulate their mRNAs. The changes of mRNAs without the alternations of their correspondent miRNAs imply that these changed mRNAs are regulated by other mechanisms. Thus, the associative assessments of miRNAs and mRNAs [14] from the brain regions that are involved in depression-related dysfunctions [34] may examine the data validation and strengthen the conclusion about the chain from stress to neuron atrophy via miRNA and mRNA/protein expression dysregulation.

We have studied molecule profiles related to major depression in depression-like mice induced by chronic unpredictable mild stress (CUMS), since the animals were thought to be the desirable system for gene analyses in the brain to reveal the molecules relevant to this disorder [6, 35] and the neuropathological changes were clearly detected in medial prefrontal cortices from CUMS-induced depression mice [34]. The gene expression was analyzed by sequencing miRNA and mRNA in the medial prefrontal cortices from CUMS-induced depression mice versus control. By the associated analyses and comparisons, we expect to figure out the signaling pathways in the medial prefrontal cortex related to stress-induced depression, in order to provide the guidelines for addressing the molecular mechanisms of major depressive disorder and for developing its therapeutic strategies.

## Methods and Materials

### Ethic issue

All experiments were conducted in accordance with the guidelines and regulations by Administration Office of Laboratory Animals at Beijing China. All experimental protocols were approved by Institutional Animal Care Unit Committee in Administration Office of Laboratory Animals at Beijing China (B10831). In terms of the living conditions for the mice under the normal life and control group, they are housed in the cages (32×16×16 cm) with free access water and food pellets under the circadian of 12 hours in the light (7:00 am to 7:00 pm) and the rest of 12 hours in the dark. Ambient temperature and relative humidity are maintained at 22±2°C and 55±5%, respectively. The standards are maintained in the specific pathogen free (SPF).

### The procedures of chronic unpredictable mild stress (CUMS)

C57BL/6J juvenile mice were used to analyze the molecular profiles associated with major depression. The male mice were used starting at postnatal days 21 for making depression model. In week one for their adaptation to the experiments, the body weight, locomotion, sucrose preference and Y-maze tests of these mice were measured to select the healthy ones for our study. The mice of showing consistent values in these measurements were separated into two groups, CUMS and control, in order to reduce the variations among these mice. The control mice lived without the following stresses.

Based on depression risk factors, such as weaknesses in cognitive function, emotional regulation, social interaction skill, circadian and stress responses [36], we used chronic stress to produce depression-like mice in the following principle. The mice lived in stressful environment, made efforts to challenge these conditions and experienced defeat outcomes, which drove them to feel cognitive and emotional inabilities and in turn to be anhedonia and low self-esteem. The procedures for the CUMS mice include their adaptation, the CUMS and the behavioral tests [34].

The stressful environments included social isolation, tilted cage, empty cage, damp sawdust cage, restraint space, white noise, strobe light and circadian disturbance [1, 5, 37–39]. Except for the social isolation, these conditions were randomly selected to treat the mice in the manners of their separations or combinations every day. These treatments were applied about 1~14 hours in durations and 1~12 hours in intervals [34]. The duration and interval were unpredictable to the mice. The CUMS was sustained for three weeks until some of these mice expressed anhedonia and low self-esteem.

### Behavior assays for major depressive disorders

Whether the CUMS-treated mice in three weeks fell into anhedonia and low self-esteem was tested in day 29~31. The sucrose preference test (SPT) and Y-maze test (YMT) were used to assess the anhedonia, and the forced swimming test (FST) was used to estimate their self-esteem [9, 40–43]. The SPT was conducted by 1% sucrose water versus water for four hours. The SPT value was presented as a ratio of the ingested sucrose water to the ingested sucrose water plus water. The YMT was performed by monitoring mouse staying in a special arm and other two arms for 2 minutes. The end of this special arm included a female mouse (named as M-arm). M-arm stay time was presented by a ratio of stay time in M-arm to that in three arms. The FST was done by recording immobile time in the water cylinder (10 centimeters in diameters and 19 centimeters in water depth at 25±1°C) for 6 minutes. To quantify the FST, immobile time and latency (a period to mouse immobility in the first time) were presented. The SPT, YMT and FST was done before and after the CUMS. Before each SPT, the mice in the CUMS and control were deprived from food and water for 3 hours to drive their intension of drinking water. In the YMT, these arms were cleaned by 70% ethanol and then water after each test to reduce the effect of odor on the test. Carefulness in these tests was taken by performing them in a quiet room, no additional stresses, same circadian circle for all mice and their adaptation in the test environment [34].

An expression of depression-like behaviors was accepted if the mice in the CUMS group showed the decreases in sucrose preference ratio and M-maze stay time as well as the increase in immobile time significantly, compared to these values during their self-control period (the first week for adaptation) and in the control group of the mice. The mice with significant changes in all of three tests were defined as CUMS-induced depression-like mice or depression-like mice. The CUMS-treated mice in 3 weeks met this criterion about 30%, implying their vulnerability to the stresses. These mice were used as depression-like mice to study the molecular profiles. Some mice without any change in the three tests are considered as resilience, i.e., their invulnerability to CUMS, which were not included in our study. The mechanisms underlying vulnerability and invulnerability to the stress are not our current topic, which will be studied in the future. As 30% of CUMS-treated mice met the depression criteria and all CUMS-treated mice did not show a change of the SPT at the end of week one, the stressful situations in our study were thought to be mild stress.

### RNA purification from medial prefrontal cortices

Twenty-four hours after the mice were surely tested to demonstrate CUMS-induced depression-like behaviors, these depression-like mice and controls were anesthetized by using Isoflurane and decapitated by guillotine. Both sides of the medial prefrontal cortices were quickly isolated and dissected on ice-cold glass slides. These cortical tissues were placed into the frozen vials that contained RNAlater RNA Stabilization Reagent (QIAGEN, Germany) at 4°C and stored at -80°C for subsequent analyses. Total RNAs from the tissue of medial prefrontal cortex in each mouse were isolated with TRIzol Reagent (Life Technologies, Carlsbad, CA, USA) based on the manufacturer instruction. Total RNA samples placed in the dried ice were delivered to Beijing Genomics Institute (BGI), China for sequencing analysis. RNA samples were done with quality-control by BGI staff using Agilent 2100 Bioanalyzer (Agilent Technologies, USA) with RNA 6000 nano Reagents Port 1. The concentrations of the total RNA, the values of RNA integrity number (RIN) and the ratios of 28S to 18S ribosomal RNA were measured. The samples with total RNA amount larger than 10 μg, the concentration larger than 200 ng/μl, the RIN larger than 8, and the ratio of 28S to 18S larger than 1.0 were selected for the construction of transcriptome and small RNA libraries, respectively.

### RNA sequencing

mRNAs were extracted from the total RNA by oligo(dT) beads. mRNAs were randomly sheared into 200 bp fragments that were reversely transcribed into the complementary DNA (cDNA) by random oligonucleotides. Upon end repair, these synthesized cDNAs were purified by using QiaQuick PCR extraction kit and ligated by sequencing adaptors. After the amplification with Illumina PCR Primer Cocktail within 15 cycles of PCR reaction, these cDNAs were size-selected and purified by agarose gel electrophoresis. cDNAs with the sizes between 200 and 300 bp were selected for the library construction.

In the meantime, the miRNA sequencing library was constructed from those total RNAs, in which low molecular weight RNAs (18–30 nt) were isolated by the polyacrylamide gel electrophoresis. 5ˊ-RNA adapter was ligated to RNAs with T4 RNA ligase. The ligated RNAs were size-fractionated and 36–50 nucleotide fractions were excised. 3ˊ-RNA adapter was subsequently ligated to the precipitated RNA by using T4 RNA ligase. The ligated RNAs were size-fractionated and the 62–75 nucleotide fraction (small RNA+adaptors) was excised. Small RNAs ligated with adaptors were subjected to RT-PCR to produce sufficient templates for the sequencing. PCR products were purified and collected by gel purification and ready for the high-throughput sequencing.

The qualities of transcriptome and small RNA libraries were assessed by using the Agilent 2100 Bioanalyzer (Agilent Technologies, CA USA). The quantity of PCR products was verified by quantitative PCR in ABI StepOnePlus Real-Time PCR System. After the quality and quantity of the prepared libraries were confirmed to be qualified, their sequencings were done by using Illumina HiseqTM 2500 platform (Illumina Inc., San Diego, CA USA). The average reading length of two libraries were about 100 bp (pair-end) and 49 bp (single-end), respectively.

### Bioinformatics for mRNA

The original image data was transferred into the sequence data by base calling, which were defined as raw data or raw reads. DynamicTrim Perl script implemented in SolexaQA package was performed to control the quality of raw sequencing data based on the following criteria. 1) Remove the reads with adapters. 2) Remove the reads in that the unknown bases were more than 10%. 3) Remove the reads with 50% of the bases with low quality score (PHRED score 5). After filtering, the remained reads were called as the "clean reads" and then mapped to mouse genome reference sequence (UCSC mm10) by using TopHat v1.0.12 which incorporated Bowtie v0.11.3 software to perform the alignments. For the alignment and mapping, the maximum of allowable mismatch was set to 3 for each read. The sole reads uniquely aligned to the genes were used to calculate gene expression level. The reads per kilo-base per million reads (RPKM) were used for gene expression and the genes with low expression level (RPKM< 0.5) were removed for further analysis.

We screened the differentially expressed genes (DEGs) based on NOIseq package method, which determined DEGs between two groups with the biological replicates. The threshold used to identify DEGs was fold-change larger than 1.5 and the diverge probability larger than 0.8. We subsequently conducted a pathway enrichment analysis of DEGs’ association with physiological or biochemical processes. In these enrichment analyses, we used the hypergeometric test implemented in the tool WebGestalt (version 2) and the canonical pathways from the Kyoto Encyclopedia of Genes and the Genomes (KEGG) database. This analysis would identify the enriched metabolic pathways or signal transduction pathways in DEGs that were compared with whole genome background. *P-*values from the hypergeometric tests were adjusted by Benjamini-Hochberg method. Those pathways with adjusted *p*-values less than 0.05 were considered to be significant enrichments.

### Bioinformatics for miRNA

49 nt-sequence tags from Hiseq sequencing were initially processed to remove adaptor sequences, low-quality reads and contaminants for the credible clean reads. To remove the reads from ncRNA (noncoding RNA), such as rRNAs (ribosomal RNAs), tRNAs (transfer RNAs), snRNAs (small nuclear RNAs), snoRNAs (small nucleolar RNAs) and repeat RNA, we aligned the reads to the Genbank database and Rfam database with blast or bowtie softwares. All of the high-quality clean reads ranging from 18–25 nt were matched to the known miRNA precursor of corresponding species in miRBase to obtain the miRNA count. The detailed criteria include: 1) align the tags to miRNA precursor in miRBase with no any mismatch, 2) based on the first criteria, the tags align to the mature miRNA in miRBase with at least 16 nt overlap allowing offsets. Those miRNAs satisfied with these criteria would be counted to get the expression of identified miRNAs. The remained reads without any annotation were used to predict the potential novel miRNAs and its stem loop structure by Miredp based on the references [44, 45]. To correct the biased results from low expression, we discarded miRNAs with read counts less than 5 in the differential expression analysis.

We used DESeq software algorithm that was based on negative binomial distribution and biology duplicate samples to compare the known or novel miRNA expression in control versus depression groups. The criteria for assigning significance included that *P*-value was less than 0.05 and the fold-change was larger than 1.5. Gene targets of differentially expressed miRNAs were predicted with four miRNA target prediction softwares, such as Targetscan, PITA, RNA22 and miRDB. To eliminate bias caused by a single database prediction, the genes predicted by more than two softwares were chosen for further analysis.

### Integrated miRNA/mRNA network analysis

To find the correlations between different expression of miRNAs and their target mRNAs, a series of bioinformatics analyses were performed. miRNAs were usually negatively correlated with their targeted mRNAs, in spite of a few exceptions [46]. To identify the potential miRNA-regulated target genes, the datasets of differentially expressed miRNAs and transcripts were integrated. We set the following criteria for the potential targets. The target mRNAs and miRNAs should be simultaneously and reversely changed in our analyses. The target mRNAs should be predicted by miRNAs at least from the two softwares of PITA, Targetscan, RNA22 and miRDB. The compliant miRNA target predictions were compared with those of DEGs from transcriptome sequencing to detect overlap. Interactive networks from the differentially expressed miRNAs and the simultaneously expressed target mRNAs were visualized by using Cytoscape software (San Diego, CA, USA).

### Quantitative RT-PCR for the validations of miRNA and mRNA

To validate the results from High-throughput sequencing, we used quantitative real-time RT-PCR (qRT-PCR) by SYBR Green technique to analyze five mRNAs and four miRNAs that were involved in different cellular functions as well as were significantly difference between control (n = 8) and depression-like mice (n = 8), in which the samples were used from those tissues for High-throughput sequencing. All gene primers produced the amplicons which spanned two exons. Each of them were located in the highly conserved coding regions and included all known alternatively spliced mRNA variants (S1 Table). Briefly, RNAs were isolated from the tissues of the medial prefrontal cortex by using Trizol method. cDNAs were synthesized for mRNA expression assays from total RNA in 10 μl volume with 2 μl PrimeScript™ RT Master Mix (TaKaRa Biotechnology Co. Ltd. Dalian China), 1 μl total RNA and 7 μl ddH_2_O. cDNAs were synthesized for miRNA expression assay from total RNA of the same sample in a 25 μl volume, including 2 μl total RNA, 2 μl reverse transcription primer (random primers for U6 rRNA and Bulge-LoopTM miRNA, RiboBio, China), 5 μl RT Buffer, 0.5 μl PrimeScript^™^ II Reverse Transcriptase (200 U/μl), 0.5 μl Rnase inhibitor (40 U/μl), 2 μl dNTP (2.5 mM) and 13 μl ddH_2_O. qRT-PCR was done by using biosystems QuantStudio 7 Flex (Life Technologies, USA). Each reaction was carried in a total volume of 20 μl including 1 μl cDNA, 10 μl SYBR Premix Ex Taq^™^ Ⅱ (TaKaRa Biotechnology Co. Ltd. Dalian, China), 0.5 μl/primer and 9 μl ddH_2_O, in which the program was set to 95°C in 5 min for pre-incubation, 40 cycles at 95°C in 5 seconds and at 60°C in 20 seconds for the annealing and amplification, as well as finally addition dissociation curve. The relative expression level of mRNAs in the tissue was normalized to an internal reference gene Beta-actin or GAPDH. The relative expression level of miRNAs in the tissue was normalized to U6 small nucleolar RNA. All qRT-PCR runs were repeated in three replications. The results were calculated with the 2^-ΔΔCt^ method.

### Dual luciferase reporter assay

The 3′-untranslated region (UTR) sequence of targeted gene was amplified and fused into the *XhoI* and *NotI* sites of a dual luciferase vector psiCHECK2, a generous gift from Dr. Xue (Institute of Biophysics, Chinese Academy of Sciences). The site-directed mutation of the detected miRNA targeting site of 3′-UTR fragment was constructed based on a guideline of QuikChange Lighting Site-Directed Mutagenesis Kit (Stratagene, La Jolla, CA, USA). For luciferase reporter detection, HEK293T cells were planted in RPMI media containing 10% fetal bovine serum at 5×10^4^ cells per well in 24-well plates. After 24 hours, these cells were co-transfected with 20 ng psiCHECK2-3’UTR wild-type or mutant reporter plasmids. In the meantime, these wells of cell culture were added by 50 nM of miRNAs mimic or miR-NC by using Lipofectamine 2000 transfection reagent (Invitrogen, Carlsbad, CA, USA). The activities of firefly and Renilla luciferase were assessed after 48 hours by using Dual-Glo® Luciferase Assay System (Promega, Cat. E2920, USA), based on manufacturer protocols. Each treatment was performed in the triplicates in three independent experiments.

### Statistical analyses

Initial processing raw data of mRNA and miRNA expression profiles were performed by using NOIseq and DESeq software algorithm, respectively. The data of the behavior tests, luciferase activity and gene analyses are presented as mean±SEM. Relationships between miRNA and its target prediction were assessed by Pearson’s correlation coefficients. Unpaired Student t-test was used to make the statistic comparison between control and depression-like mice. *P*<0.05 is considered statistically significant.

## Results

### Chronic unpredictable mild stress induces the mice to express depression-like behaviors

**Fig 1:**
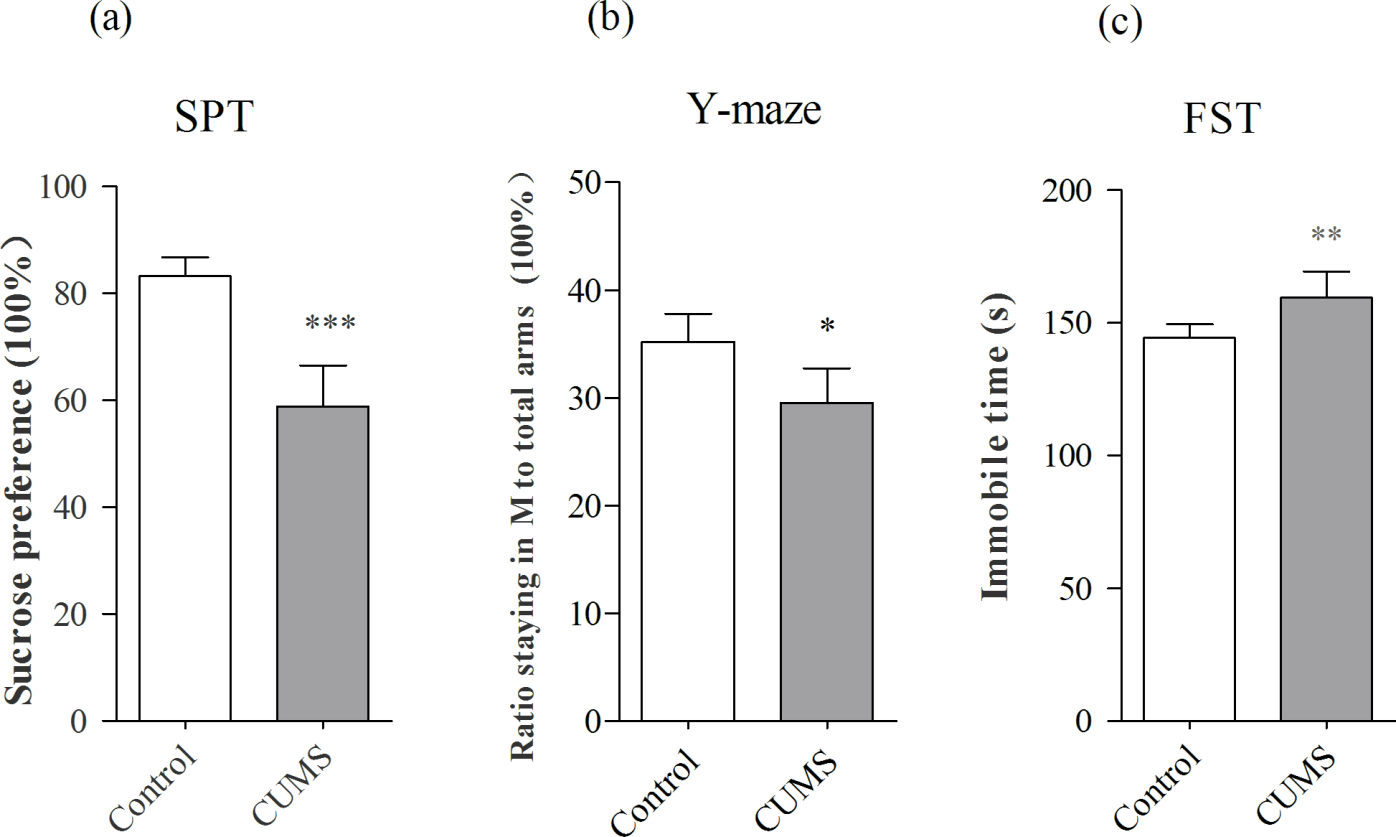
Chronic unpredictable mild stress (CUMS) leads the mice to express depression-like behaviors. Mice were subjected to the adaptation for a week, the CUMS for three weeks and the behavioral tests in three days. Behavior tests showed the significant decreases in sucrose preference (a) and the ratios of stay time in M-arm to stay time in three arms (b) as well as the increase in immobile time (c) from CUMS-induced depression mice (n = 10) and controls (n = 11). The results are expressed as mean ± SEM. *p<0.05, **p<0.01, ***p<0.001.

The mice were treated by CUMS or control for three weeks and their mood states were assessed by the tests of sucrose preference (SP), Y-maze (YM) and forced swimming (FS), where the procedures were given in our previous study [34]. The mice with significant changes in all of three tests were defined as CUMS-induced depression-like mice, or depression-like mice. 30% of the CUMS-treated mice met the criteria of depression state. In the mice of expressing the significant changes in all of these tests, the SPT values are 59.58±2.2% in CUMS-treated mice (n = 10) and 83.62±1% in control mice (n = 11, Fig 1A). The values from CUMS-treated mice versus controls are statistical difference (*p*<0.001, unpaired student t-test). The ratios of stay time in the M-arm to stay time in the total arms are 29.5±1.6% in CUMS-treated mice (n = 10) and 35.2±1.3% in controls (n = 11, *p*<0.05, unpaired student t-test; Fig 1B). The values of immobile time in the FST are 159.6±3.3 seconds in CUMS-treated mice (n = 10) and 144.4±1.8 seconds in controls (n = 11, *p*<0.01, unpaired student t-test; Fig 1C). The mice that show the significant changes in all of these values are thought as depression-like mice, which are used for studying molecular profiles.

As neuronal dysfunction in the medial prefrontal cortex is associated to depression-like behaviors [34], we investigated its molecular mechanisms by sequencing miRNAs and mRNAs to quantify their expression levels in this cortical area from depression mice and controls.

### Overall qualities of RNA-Sequencing dataset

High-throughput RNA-sequencing was used to measure transcriptome and microRNA expression profiles in each sample of the medial prefrontal cortices from depression-like mice versus control mice. RNAs from 45123294 to 45175652 raw sequence reads about 100 bp were obtained from mRNA library Illumina sequencing (two depression-like libraries and two control libraries). After filtering the reads that contained N with adaptor sequence and low quality, 4.5 GB clean reads from each library were generated and mapped, which were about 78.84~79.62% of the total reads from the mouse genome (UCSC mm10) equivalently for all samples (S2 Table). In addition, the totals about 14297163~14962143 raw sequence reads were produced in the small RNA library. After filtering and trimming the reads with low quality and adaptor, the totals of clean small RNA reads about 13838056~14487369 were obtained (S3 Table). The distribution of the clean small RNA reads varied in a range of 10 to 44 nucleotides each library. The most abundant lengths were 22 nucleotides (S1 Fig). All high-quality clean reads larger than 18 nucleotides were mapped to the mouse genome. The genome-matched reads were divided into the different categories of small RNAs according to their biogenesis and annotation (S2 and S3 Figs). The most abundant RNA category from each library was miRNA. The high qualities of transcriptome and small RNA sequencing data were used for further analysis.

### The changes of mRNA in the medial prefrontal cortices from depression-like mice versus control

mRNAs in the medial prefrontal cortex (mPFC) were quantified by sequencing total RNAs. We computed RPKM values for those genes. Genes with lower expression level (RPKM<0.5) were removed, such that 17,778 mRNAs were left for differential expression analyses with NOISeq. After mapping the reads referred to the mouse genome, these 17,778 mRNAs from clean read sequences with high quality included the 8181 upregulated mRNAs and the 9381 downregulated mRNAs by the comparisons between CUMS-induced depression and control mice. The expression profile of mRNAs is presented in Table 1 if their expressions alter above 1.5 fold in depression-like mice versus controls (probability≥0.8), which is a criterion to make sure gene expression alternations [32, 33]. 40 mRNAs are significantly downregulated in the mPFC from CUMS-induced depression mice as well as 10 mRNAs are upregulated (Table 1). The decreased expressions of mRNAs in CUMS-induced depression mice include Slc6a11, Hap1, Gad1, Gad2, Gng4, Slc32a1, Doc2g, Slc32a1, Magel2, Prkcd, Ngfr, Dusp1, Th, Itih3, Cacna2d2, Arc, Mbp, Peg10, Fos, and so on. Based on the bioinformatics of mRNA-guided protein translation (KEGG database), the downregulations of these genes and their translated proteins in Table 1 are associated to the dysfunction of the following signaling pathways and processes in the mPFC neurons from depression-like mice, such as the GABAergic synapses, dopaminergic synapses, synaptic vesicle recycling, amphetamine/morphine addictions, neurotrophin signaling, MAPK signaling and chemokine-cytokine receptor signaling (Table 2). Thus, the GABAergic synapses, dopaminergic synapses, reward circuits, axon growth and neural-immune process in the medial prefrontal cortex appear dysfunctional for the pathogenesis of major depression. It is noteworthy that the genes, such as Hbb-b1, Nr1d1 and so on, are upregulated in the CUMS-induced depression mice, though their roles in major depression are unclear.

**Table 1:** mRNAs with differential expression over 1.5 folds and their characteristics.

Symbol	GeneID	Means-Control	Means-CUMS	Fold change (CUMS/Control)	Probability	Chromosomal Map	Description
Mbp	17196	1167.25	774.21	0.663277	0.808471	18 E2-E4	myelin basic protein
Gad1	14415	158.22	104.29	0.659166	0.808415	2 D	glutamate decarboxylase 1
Htra1	56213	95.96	61.69	0.642924	0.814490	7 F3	HtrA serine peptidase 1
Slc6a11	243616	97.57	61.69	0.632232	0.819693	6 E3	neurotransmitter transporter, GABA
Hap1	15114	115.88	73.03	0.630221	0.822365	11 D	huntingtin-associated protein 1
Mobp	17433	82.22	51.47	0.626003	0.821802	9 F4	myelin-associated oligodendrocytic basic protein
Cldn11	18417	93.97	58.82	0.625964	0.823124	3 A3	claudin 11
Zcchc12	72693	67.07	41.91	0.624823	0.819159	X A3.3	zinc finger, CCHC domain containing 12
Sparc	20692	144.56	89.25	0.617425	0.829846	11 B1	secreted acidic cysteine rich glycoprotein
Nrsn2	228777	74.56	45.84	0.614833	0.824418	2 G3	neurensin 2
Dusp1	19252	35.145	21.27	0.605349	0.815474	17 A2-C	dual specificity phosphatase 1
Gng4	14706	36.19	21.61	0.597044	0.819327	13 A1	guanine nucleotide binding protein
Npas4	225872	25.01	14.85	0.593762	0.810412	19 A	neuronal PAS domain protein 4
Cryab	12955	64.66	38.07	0.588849	0.833305	9 A5.3	crystallin, alpha B
Slc32a1	22348	76.71	44.67	0.582285	0.838340	2	GABA vesicular transporter
Gpx3	14778	15.01	8.37	0.557776	0.800456	11	glutathione peroxidase 3
Gad2	14417	43.93	23.94	0.545072	0.843796	2 A3	glutamic acid decarboxylase 2
Calb2	12308	36.07	19.11	0.529868	0.844246	8 E1	calbindin 2
Cacna2d2	56808	22.5	11.08	0.492444	0.851783	9 F1	calcium channel, voltage-dependent, alpha 2/delta subunit 2
Fos	14281	46.38	22.25	0.479789	0.872764	12 D2	FBJ osteosarcoma oncogene
Arc	11838	184.07	88.04	0.478323	0.882017	15 D3	activity regulated cytoskeletal-associated protein
Agxt2l1	71760	9.65	4.47	0.462973	0.813787	3 G3	alanine-glyoxylate aminotransferase 2-like 1
Ngb	64242	10.68	4.79	0.44876	0.828355	12	neuroglobin
Cyr61	16007	10.64	4.56	0.42837	0.834683	3 H2	cysteine rich protein 61
Itih3	16426	19	7.78	0.409737	0.868883	14	inter-alpha trypsin inhibitor, heavy chain 3
Zic1	22771	17.51	6.91	0.394804	0.869614	9 E3.3	zinc finger protein of the cerebellum 1
Peg10	170676	10.83	3.88	0.358726	0.854792	6 A1	paternally expressed 10
Nts	67405	9.83	3.15	0.320956	0.864130	10 D1	neurotensin
Th	21823	7.78	2.44	0.313625	0.847789	7 F5	tyrosine hydroxylase
Doc2g	60425	12.95	4.04	0.312355	0.881792	19 A	double C2, gamma
Ecel1	13599	17.63	5.44	0.308565	0.893323	1 D	endothelin converting enzyme-like 1
Baiap3	545192	24.13	7.34	0.30433	0.901817	17 A3.3	BAI1-associated protein 3
Ngfr	18053	5.1	1.49	0.293137	0.807993	11 D	nerve growth factor receptor
Agt	11606	12.24	3.46	0.282564	0.884689	8 E2	angiotensinogen
Dlk1	13386	10.92	2.76	0.253205	0.883226	12 E-F1	delta-like 1 homolog
Prkcd	18753	17.22	4.3	0.249637	0.906626	14 B	protein kinase C, delta
A230065H16Rik	380787	9.24	2.03	0.219697	0.885308	12 F1	RIKEN cDNA A230065H16 gene
Magel2	27385	3.79	0.76	0.200528	0.804534	7 C	melanoma antigen, family L, 2
Igsf1	209268	7.05	1.31	0.185684	0.877995	X A5	immunoglobulin superfamily, member 1
Tmem254c	100039192	5.26	0.96	0.18251	0.850405	14 A3	transmembrane protein 254c
S100a8	20201	0.37	4.47	11.92	0.872427	3 F1-F2	S100 calcium binding protein A8
Tmem254b	100039257	3.74	9.44	2.524064	0.835583	14 A3	transmembrane protein 254b
Gm129	229599	8.29	17.38	2.097105	0.849055	3 F2.1	predicted gene 129
Rs5-8s1	790956	9.10	18.62	2.045579	0.847030	17	5.8S ribosomal RNA
Hba-a2	110257	62.98	125.58	1.993966	0.875267	11	hemoglobin alpha, adult chain 2
Beta-s	100503605	119.82	209.28	1.746662	0.846243	7	hemoglobin subunit beta-1-like
Hba-a1	15122	125.14	203.3	1.624516	0.830999	11 A4	hemoglobin alpha, adult chain 1
Hbb-b1	15129	32.64	52.95	1.622396	0.819468	7 E3	hemoglobin, beta adult major chain
Nr1d1	217166	54.85	86.95	1.585232	0.818652	11 D	nuclear receptor subfamily 1, group D, member 1
Flot2	14252	40.20	61.47	1.529039	0.801131	11 B5	flotillin 2

**Table 2:** Signaling pathways identified by KEGG function analysis based on DEGs data.

Pathway	DEGs with pathway annotation (45)	All genes with pathway annotation (16857)	Contributing Genes	Rich Factor	P-value^a^	Pathway ID
GABAergic synapse	6 (13.33%)	118 (0.7%)	Slc6a11(GAT3), Hap1,Gad2, Gng4,Slc32a1(VGAT), Gad1	0.050847	0.000001	ko04727
Neurotrophin signaling pathway	4 (8.89%)	242 (1.44%)	Magel2, Prkcd, Ngfr,Peg10	0.016529	0.003892	ko04722
MAPK signaling pathway	5 (11.11%)	403 (2.39%)	Dusp1, Itih3, Cacna2d2, Fos, Peg10	0.012407	0.004241	ko04010
Dopaminergic synapse	3 (6.67%)	193 (1.14%)	Fos, Gng4,Th	0.015544	0.014738	ko04728
Amphetamine addiction	3 (6.67%)	126 (0.75%)	Arc,Fos,Th	0.023810	0.004602	ko05031
Chemokine signaling pathway	4 (8.89%)	281 (1.67%)	Gm21586,Prkcd,Gm20878,Gng4	0.014235	0.006585	ko04062
Cytokine-cytokine receptor interaction	4 (8.89%)	348 (2.06%)	Gm20878,Gm21586,Ngfr,Peg10	0.011494	0.013673	ko04060
Synaptic vesicle cycle	2 (4.44%)	119 (0.71%)	Doc2g,Slc32a1	0.016807	0.040170	ko04721
Renin-angiotensin system	2 (4.44%)	26 (0.15%)	Agt,Peg10	0.076923	0.002174	ko04614
Morphine addiction	2 (4.44%)	113 (0.67%)	Gng4,Slc32a1	0.017699	0.036569	ko05032
Chagas disease (American trypanosomiasis)	2 (4.44%)	139 (0.82%)	Fos,Peg10	0.014388	0.053078	ko05142
African trypanosomiasis	5 (11.11%)	60 (0.36%)	Beta-s,Hbb-b1,Hba-a2,Hba-a1,Peg10	0.083333	0.000001	ko05143
Malaria	4 (8.89%)	80 (0.47%)	Beta-s,Hbb-b1,Hba-a2,Hba-a1	0.050000	0.000060	ko05144
Taurine and hypotaurine metabolism	2 (4.44%)	14 (0.08%)	Gad2,Gad1	0.142857	0.000621	ko00430
Type I diabetes mellitus	3 (6.67%)	86 (0.51%)	Gad2,Gad1,Peg10	0.034884	0.001558	ko04940
Alanine, aspartate and glutamate metabolism	2 (4.44%)	41 (0.24%)	Gad2,Gad1	0.048780	0.005348	ko00250
beta-Alanine metabolism	2 (4.44%)	46 (0.27%)	Gad2,Gad1	0.043478	0.006694	ko00410
Intestinal immune network for IgA production	2 (4.44%)	59 (0.35%)	Gm20878,Gm21586	0.033898	0.010825	ko04672
Taurine and hypotaurine metabolism	2 (4.44%)	14 (0.08%)	Gad2,Gad1	0.142857	0.000621	ko00430
Butanoate metabolism	2 (4.44%)	53 (0.31%)	Gad2,Gad1	0.037736	0.008807	ko00650

^a^ *P-*values from the hypergeometric tests were adjusted by Benjamini-Hochberg method.

In order to confirm the data about gene downregulation and upregulation, we ran quantitative RT-PCR (qRT-PCR) from the tissues that had be used for the mRNA sequencing. The expressions of Hbb-b1 (S4A Fig) and Nr1d1 (S4B Fig) are raised, as well as the expressions of Gad1 (S4C Fig), Mbp (S4D Fig) and Slc6a11 (S4E Fig) are decreased in CUMS-induced depression mice, compared to the control mice (*p*<0.01). The consistent results achieved by mRNA sequencing and qRT-PCR support the validation of our study.

The level of mRNAs in the cells is affected by miRNAs, through which the bindings of miRNAs with their dicers degrade mRNAs and weaken their translations [47–50]. If the downregulated mRNAs in the mPFC from depression mice are caused by miRNAs, their correspondent miRNAs will be upregulated. To test this hypothesis and validate our data about mRNA changes, we analyzed the changes of miRNAs by their sequencings in mPFCs from depression-like mice versus controls.

### The changes of miRNA in the medial prefrontal cortices from depression-like mice versus control

**Fig 2:**
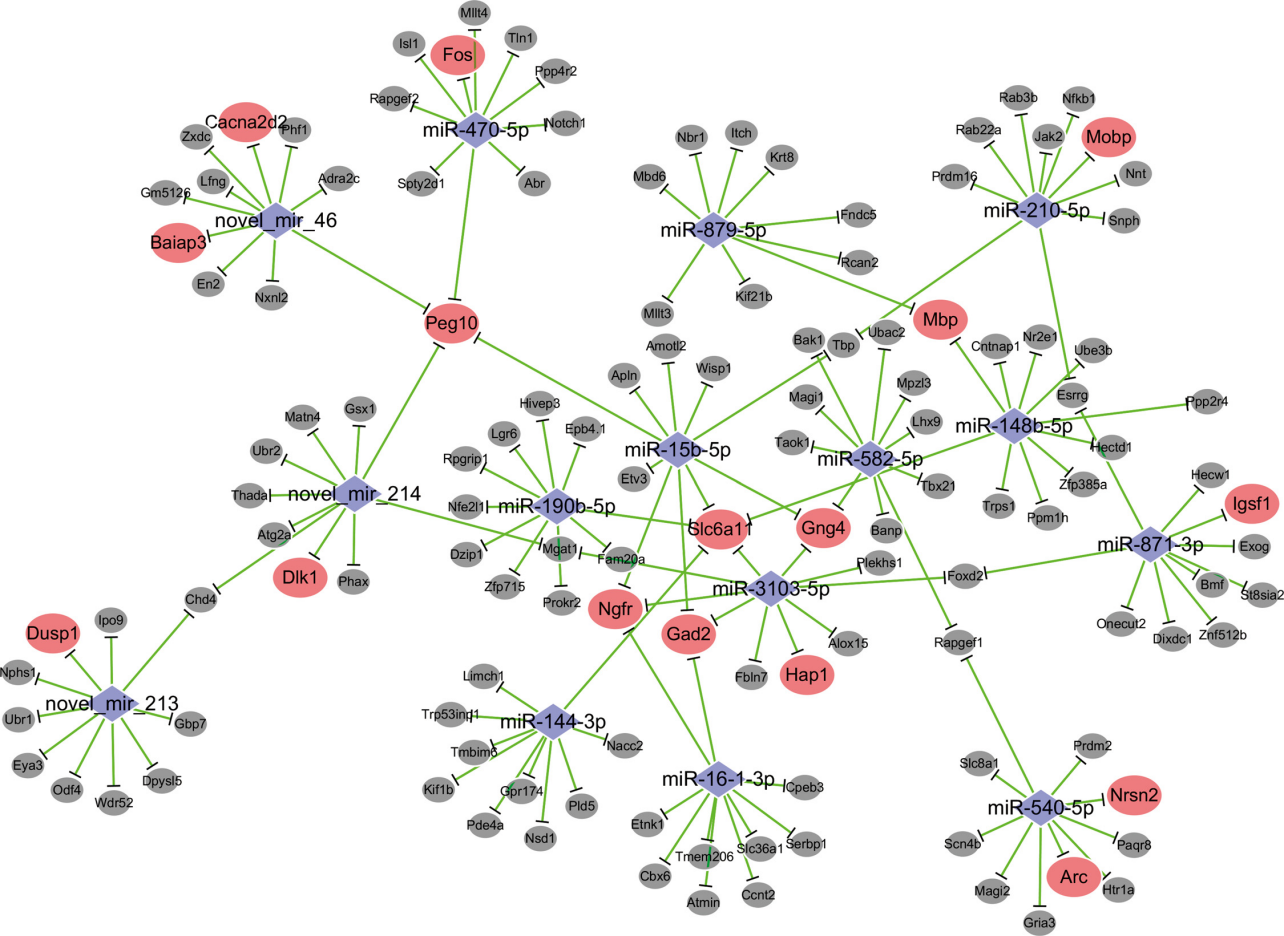
MicroRNA-mRNA network. microRNA/mRNA networks were constructed between the fifteen upregulated miRNAs and 150 overlapped mRNAs with using transcriptome expression data and predicted target genes from Targetscan, PITA, RNA22 and miRDB databases. Blue symbols present the elevated expression of miRNA in CUMS-induced depression mice. Red symbols present the downregulated genes that are miRNA-predicted and -targeted in CUMS-induced depression mice. Gray symbols show mRNAs that are slightly downregulated. Their negative relationship was represented by the green bars.

The expression profile of miRNAs is presented in Table 3 if their expressions change above 1.5 fold in all of the depression-like mice versus controls. These upregulated miRNAs include certain known miRNAs (mmu-miR-148b-5p, mmu-miR-879-5p, mmu-miR-144-3p, mmu-miR-540-5p, mmu-miR-582-5p, mmu-miR-15b-5p, mmu-miR-210-5p, mmu-miR-871-3p, mmu-miR-3103-5p, mmu-miR-16-1-3p, mmu-miR-470-5p, mmu-miR-190b-5p, mmu-miR-384-5p and mmu-miR-490-5p), as well as some novel miRNAs (novel_mir_46, novel_mir_214 and novel_mir_213) with their stem loop structures by Miredp (S3 Fig). Their predicted target mRNAs well match the measures by mRNA sequencing based on the databases (PITA, Targetscan, RNA22 and miRDB) about the complex interactions between miRNAs and mRNAs (S4 Table). Table 4 shows the alternated miRNAs and their predicted-target mRNAs. Table 5 shows the altered mRNAs and their correspondent miRNAs. Interactive networks about the upregulated miRNAs and 150 overlapped mRNAs, which were based on transcriptome expression data and predicted target genes from four databases, were made in the Cytoscape (Fig 2). By reading Tables 1 and 3–5, we can find that the downregulations of mRNAs and the upregulations of miRNAs are well matched. The consistent results by associatively sequencing mRNAs and miRNAs validate our analyses and strengthen our conclusion.

**Table 3:** miRNAs with quantitative change over 1.5 folds and their characteristics.

Known miRNA	Accession no	Means; Control	Means; CUMS	Fold change (CUMS/Control)	p-value	Chromosomal location (mouse)	Seed sequence
mmu-miR-148b-5p	MIMAT0017036	115.89	175.45	1.51	0.000393	chr15: 103285125–103285221 [+]	5'-aaguucu-3'
mmu-miR-879-5p	MIMAT0004842	719.47	1442.29	2.00	0.047942	chr5: 9375704–9375779 [+]	5'-gaggcuu-3'
mmu-miR-144-3p	MIMAT0000156	78.92	121.24	1.54	0.005340	chr11: 78073005–78073070 [+]	5'-acaguau-3'
mmu-miR-540-5p	MIMAT0004786	68.46	120.24	1.76	0.000003	chr12: 109586080–109586146 [+]	5'-aaggguc-3'
mmu-miR-582-5p	MIMAT0005291	73.19	111.12	1.52	0.001605	chr13: 109324744–109324824 [+]	5'-uacaguu-3'
mmu-miR-15b-5p	MIMAT0000124	63.87	100.30	1.57	0.007213	chr3: 69009772–69009835 [+]	5'-agcagca-3'
mmu-miR-7240-5p	MIMAT0028448	60.81	99.88	1.64	0.010544	chr8: 70798135–70798192 [+]	5'-uggagag-3'
mmu-miR-210-5p	MIMAT0017052	59.39	92.52	1.55	0.000017	chr7: 141221384–141221493 [–]	5'-gccacug-3'
mmu-miR-871-3p	MIMAT0017265	46.85	73.27	1.56	0.000011	chrX: 66810428–66810504 [–]	5'-gacuggc-3'
mmu-miR-3103-5p	MIMAT0014937	34.73	60.44	1.74	0.000865	chr7: 128288369–128288435 [–]	5'-gagggag-3'
mmu-miR-16-1-3p	MIMAT0004625	26.27	41.73	1.59	0.001608	chr14: 61631880–61631972 [–]	5'-caguauu-3'
mmu-let-7a-1-3p	MIMAT0004620	17.70	32.60	1.84	0.022997	chr13: 48538179–48538272 [–]	5'-uauacaa-3'
mmu-let-7c-2-3p	MIMAT0005439	17.70	32.60	1.84	0.002300	chr15: 85706603–85706697 [+]	5'-uauacaa-3'
mmu-miR-470-5p	MIMAT0002111	11.34	24.42	2.15	0.033824	chrX: 66813951–66814025 [–]	5'-ucuugga-3'
mmu-miR-218-2-3p	MIMAT0005444	7.37	17.53	2.37	0.016864	chr11: 35616816–35616925 [+]	5'-augguuc-3'
mmu-miR-190b-5p	MIMAT0004852	7.86	13.89	1.76	0.000257	chr3: 90070020–90070099 [+]	5'-gauaugu-3'
mmu-miR-6240	MIMAT0024861	5.41	15.42	2.85	0.020825	chr5: 114851948–114852064 [+]	5'-caaagca-3'
mmu-miR-3083-3p	MIMAT0014875	9.47	25.28	2.67	0.004859	chr17: 26948056–26948119 [–]	5'-ccgaaac-3'
mmu-miR-384-5p	MIMAT0004745	519.42	298.63	0.57	0.010486	chrX: 105344282–105344369 [–]	5'-guaaaca-3'
mmu-miR-1264-3p	MIMAT0014803	62.35	32.95	0.53	0.000058	chrX: 147010601–147010686 [+]	5'-aaaucuu-3'
mmu-miR-217-5p	MIMAT0000679	42.51	9.24	0.22	0.001059	chr11: 28763728–28763835 [+]	5'-acugcau-3'
mmu-miR-490-5p	MIMAT0017261	80.02	53.61	0.67	0.000251	chr6: 36421742–36421825 [+]	5'-cauggau-3'
novel_mir_46		48.68	108.08	2.22	0.026944	chr2:181459599–181459621	5'-agcgggc-3'
novel_mir_214	Only in CUMS		15.34	Inf	0.026173	chr4:41858835–41858855	5'-actgcct-3'
novel_mir_213	Only in CUMS		13.95	Inf	0.043573	chr3:121770702–121770723	5'-ctgagcc-3'
novel_mir_10	Only in control	32.27		Inf	0.000021	chr11:105913296–105913317	5'-ttgaaag-3'
novel_mir_39	Only in control	19.03		Inf	0.004927	chr1:25228756–25228777	5'-gctggac-3'
novel_mir_128	Only in control	17.31		Inf	0.008822	chr17:26948060–26948081	5'-aggctgg-3'
novel_mir_54	Only in control	14.13		Inf	0.035147	chr4:41858835–41858857	5'-actgcct -3'

**Table 4:** The changed miRNAs predict target mRNAs.

miRNAs	The predicted target mRNAs that match DEGs in transcriptome *
mmu-miR-148b-5p↑	Slc6a11(GAT-3)↓, Mbp↓
mmu-miR-879-5p↑	Slc6a11(GAT-3)↓, Mbp↓
mmu-miR-144-3p↑	Slc6a11(GAT-3)↓
mmu-miR-540-5p↑	Arc↓, Nrsn2↓
mmu-miR-582-5p↑	Gng4↓
mmu-miR-15b-5p↑	Slc6a11(GAT-3)↓, Gad2↓, Ngfr↓, Gng4↓, Peg10↓
mmu-miR-210-5p↑	Mobp↓
mmu-miR-871-3p↑	Igsf1↓
mmu-miR-3103-5p↑	Slc6a11↓(GAT-3), Hap1↓, Gng4↓, Ngfr↓, Gad2↓
mmu-miR-16-1-3p↑	Gad2↓, Ngfr↓
mmu-miR-470-5p ↑	Fos↓, Peg10↓, Nr1d1↓
mmu-miR-190b-5p↑	Slc6a11(GAT-3)↓
novel_mir_46 ↑	Baiap3↓, Cacna2d2↓, Peg10↓
novel_mir_214 ↑	Dlk1↓, Peg10↓
novel_mir_213↑	Dusp1↓
novel_mir_128↓	Flot2↑

* Note: The target mRNAs should be predicted by more than two softwares of PITA, Targetscan, RNA22 and miRDB, and then overlapped to DEGs in transcriptome sequencing.

**Table 5:** The changed mRNAs are regulated by miRNAs.

mRNAs	microRNAs that are predicted to act onto target mRNAs by more than 2 of PITA, Targetscan, RNA22 and miRDB
Slc6a11↓	mmu-miR-879-5p↑, mmu-miR-148b-5p↑, mmu-miR-144-3p↑, mmu-miR-15b-5p↑, mmu-miR-3103-5p↑, mmu-miR-190b-5p↑
Gad2↓	mmu-miR-15b-5p↑, mmu-miR-3103-5p↑, mmu-miR-16-1-3p↑
Gng4↓	mmu-miR-582-5p↑, mmu-miR-15b-5p↑, mmu-miR-3103-5p↑
Hap1↓	mmu-miR-3103-5p↑
Ngfr↓	mmu-miR-15b-5p↑, mmu-miR-3103-5p↑, mmu-miR-16-1-3p↑
Peg10↓	mmu-miR-15b-5p↑, mmu-miR-470-5p↑,novel_mir_46 ↑, novel_mir_214↑
Dusp1↓	novel_mir_213↑
Cacna2d2↓	novel_mir_46↑
Fos↓	mmu-miR-470-5p↑
Mbp↓	mmu-miR-148b-5p↑, mmu-miR-879-5p↑, mmu-miR-144-3p↑
Igsf1↓	mmu-miR-871-3p↑
Mobp↓	mmu-miR-210-5p↑
Nrsn2↓	mmu-miR-540-5p↑
Arc↓	mmu-miR-540-5p ↑
Dlk1↓	novel_mir_214↑
Baiap3↓	novel_mir_46 ↑
Flot2 ↑	novel_mir_128↓

Note: ↑ indicates up-regulation in the tissue of PFC from depression-like mice versus control mice, whereas ↓ represents down-regulation.

In order to validate the finding by miRNA sequencing analysis, four upregulated miRNAs (mmu-miR-879-5p, mmu-miR-582-5p, mmu-miR-144-3p, mmu-miR-15b-5p) were selected for doing qRT-PCR. Consistent with high-throughput sequencing, these miRNAs are significantly increased in qRT-PCR from CUMS-induced depression mice, compared to control mice (S4F–S4I Fig). Their predicted target mRNAs match the actually downregulated mRNA in transcriptome. The consistent results from the analyses by miRNA sequencing and qRT-PCR also support the validation of our study.

### GAT-3 mRNA is the targets of miRNA-15b-5p and miRNA-879-5p

To validate the silico prediction (Tables 4 and 5), we selected miRNA-15b-5p and miRNA-879-5p to examine whether they targeted GAT3 by qRT-PCR and dual luciferase reporter assay. There are inverse correlations between these miRNAs and GAT3 from qRT-PCR analysis (S5A and S5B Fig). Furthermore, in dual luciferase report assay, we constructed luciferase reporter plasmids, which contained the wild-type or mutant 3′-UTRs of the predicted binding sites of the miRNAs in GAT-3. These reporter constructs were transfected into HEK293T cells. After miRNAs mimics or their negative controls are applied, the relative activities of luciferase reporter for the 3′-UTR of GAT-3 mRNA are significantly lowered by the mimics of miRNA-15b-5p (S5C Fig) or miRNA-879-5p (S5D Fig), which are reversed by mutating the binding sites of miRNA-15b-5p and miRNA-879-5p (S5C and S5D Fig). These results suggest that GAT-3 mRNA is the direct target of miR-15b-5p and miRNA-879-5p, which grants our bioinformatics analyses for the prediction of miRNA target genes.

### The comparisons among our data with other studies

The expressional alternations of depression-related miRNAs and mRNAs have been reported in current years [51–59]. From such data, some discrepancies are found, which may be due to these studies by using different experiment models, brain areas, animal species and/or analysis approaches. By data comparisons, we expect to figure out the direction of future studies based on their consistent results and to identify the model-specific or cortex-specific genes based on their differences. S5 Table demonstrates this comparison of the involved miRNAs in context of the experiment subjects, brain regions and analysis approaches.

## Discussion

By sequencing both mRNAs and miRNAs to merit their expression levels, we have analyzed the quantitative changes of total mRNAs and miRNAs in the medial prefrontal cortices from CUMS-induced depression mice versus control mice. mRNAs are downregulated that encode the neural processes, such as GABAergic synapse, dopaminergic synapse, synaptic vesicle cycle, axon growth, protein phosphorylation and amphetamine/morphine addiction. In the meantime, miRNAs that target such mRNAs are upregulated. The consistent results from sequencing mRNAs and miRNAs (Tables 1 and 3) as well as quantitative RT-PCR (S4 Fig) strengthen the results from our analyses to be confident. Furthermore, consistent results achieved from qRT-PCR and dual luciferase reporter assays strengthen bioinformatics analysis of miRNA target prediction (S5 Fig). It is noteworthy that a few of miRNAs and mRNAs change toward the same direction, which are not presented in current presentation (bold fonts in S4 Table).

The attenuations of GABAergic neurons and synaptic transmission in the medial prefrontal cortex are indicated by the morphological and functional studies [34, 60–66]. These indications are supported by our analyses by sequencing mRNA and miRNAs (Tables 1–3 and Fig 2). As GABAergic neurons regulate the coordination and encoding ability of excitatory neurons [67–70], the attenuation of GABAergic neurons leads to the dysfunction of neuronal networks in the medial prefrontal cortex. Furthermore, our analyses indicate the attenuation of dopaminergic synapse, axonal growth and synaptic vesicle recycle. As we known, major depressive disorders are characterized as anhedonia and low self-esteem, which may result from the dysfunction of the neurons and synapses in the reward circuits, such as dopaminergic synapses and the degeneration of the axons in the medial prefrontal cortex and nucleus accumbens. This indication supports a current view about the deficiency of the reward circuits in psychological diseases, such as major depression, bipolar disorder and schizophrenia [71, 72].

Previous studies indicate a downregulation in the genes of synapse-related elements, GABAergic synapses and myelin-associated protein in depression-like rats or mice and depression patients, where the analytical methods of quantitative RP-PCR, microarray or *in situ* hybridization are used [52, 53, 56, 73]. This indication is also showed in our quantitative analysis by sequencing mRNAs from CUMS-induced depression mice. In addition to consistent indication, our analysis further implies the dysfunctions of dopaminergic synapses and protein phosphorylation signaling pathway, which bring new insight into molecular mechanism underlying major depressive disorders. This new finding may result from the application of mRNA sequencing method that is advanced to date.

Through sequencing miRNAs for their quantifications, we show that some known miRNAs (miR-148b-5p, miR-879-5p, miR-144-3p, miR-540-5p, miR-582-5p, miR-15b-5p, miR-210-5p, miR-871-3p, miR-3103-5p, miR-16-1-3p, miR-470-5p, miR-190b-5p, miR-384-5p and miR-490-5p) are upregulated in CUMS-induced depression mice (Table 3), which degrade mRNAs listed in Table 1. In other words, the analysis from miRNA sequencing is consistent to the analysis from mRNA sequencing. Furthermore, the upregulation in some of these miRNAs is indicated in previous analyses by quantitative RT-PCR, *in situ* hybridization or microarray in rats, mice or human beings (S4 and S5 Tables and [51, 54, 55, 57–59]). Thus, these consistencies indicate the validation of our study. On the other hand, our analysis shows that some novel miRNAs (novel_mir_46, novel_mir_214 and novel_mir_213) are upregulated in depression-like mice (Table 3), which in turn downregulate mRNAs that encode the calcium channels and intracellular Ca^2+^ signaling pathways. These alternations may lead to the neuron and synapse atrophies in major depressive disorders as the overload of intracellular Ca^2+^ and the activation of its signaling pathway lead to neuronal death [74–76].

It is noteworthy that certain differences can be read out between our analysis (Tables 1 and 3) and previous studies (S5 Table; [51–59]). These inconsistences may be due to the studies in these reports by using the different experimental models, brain regions and analysis methods. Moreover, many miRNAs in primate-specificity may be involved in these differences [77]. The quantitative alternations of miRNAs in some reports without the alternation of their targeted mRNAs in others may be caused by the following reasons. The changes in miRNAs may not reach the threshold to regulate their targeted mRNAs. Some miRNAs may alter with their targeted mRNAs in the same direction [46]. circRNAs act as miRNA sponges and positive regulators of miRNA-targeted genes [78–81]. On the other hand, the quantitative changes of mRNAs without the alteration of their correspondent miRNAs imply that the altered mRNAs are likely regulated by other epigenetic mechanisms, such as DNA methylation and repressive histone modification in the promoters. Therefore, the associated assessments of miRNAs and mRNAs by their sequencings in the brain regions from the mice with depression-related dysfunction [34] may examine the data validation and strengthen the conclusion. The consistence between our data and others should shed light on the future direction to study the pathological mechanisms and to explore therapeutic strategies for major depressive disorders [82].

## Supporting Information

S1 Fig Length distribution of small RNA library for control and CUMS groups. (PDF)

S2 Fig Small RNA categories annotation for control and CUMS groups. (PDF)

S3 Fig Stem loop structure and predicted mature sequence. (PDF)

S4 Fig The validation of differentially expressed mRNAs and miRNAs in the medial prefrontal cortex (mPFC) from CUMS-induced depression mice versus control mice. (PDF)

S5 Fig The miRNAs targeted mRNAs are validated by qRT-PCR and Luciferase reporter assay. (PDF)

S1 Table qRT-PCR prime information. (DOCX)

S2 Table Filtering transcriptome raw data and alignment. (DOCX)

S3 Table Filtering small RNA library raw data and quality control. (DOCX)

S4 Table The network of microRNA and mRNA. (XLSX)

S5 Table miRNA changes in psychiatric disorders from other studies in comparison with our data. (DOCX)
