# Supplementary material for: Molecular Mechanism for Stress-Induced Depression Assessed by Sequencing miRNA and mRNA in Medial Prefrontal Cortex
Source: PLoS One. 2016 Jul 18;11(7):e0159093. doi: 10.1371/journal.pone.0159093 (PMC4948880; doi:10.1371/journal.pone.0159093)
Supplement: S1 Fig — (PDF) [file pone.0159093.s001.pdf]

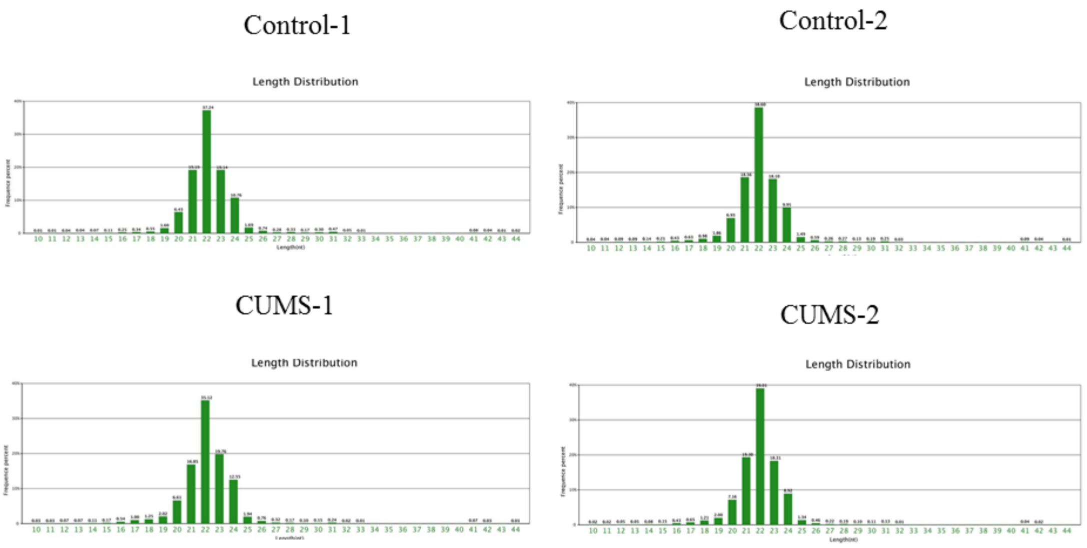

S1 Fig. Length distribution of small RNA library for control and CUMS groups. The distribution of nucleotide lengths of clean small RNA reads varied from 10 to 44 nucleotides in each library and the most abundant length was 22 nucleotides.
