# Supplementary material for: Molecular Mechanism for Stress-Induced Depression Assessed by Sequencing miRNA and mRNA in Medial Prefrontal Cortex
Source: PLoS One. 2016 Jul 18;11(7):e0159093. doi: 10.1371/journal.pone.0159093 (PMC4948880; doi:10.1371/journal.pone.0159093)
Supplement: S2 Fig — (PDF) [file pone.0159093.s002.pdf]

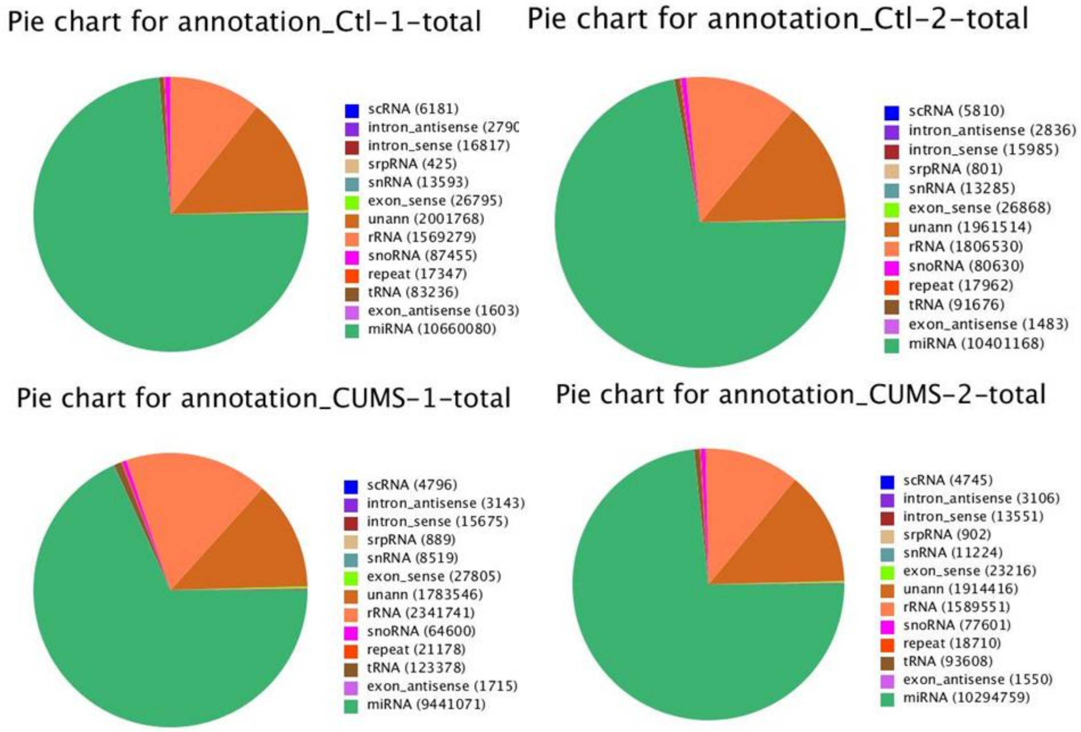

S2 Fig. Small RNA categories annotation for control and CUMS groups. All of the high-quality clean reads larger than 18 nucleotides were mapped to the mouse genome and these genome-matched reads were divided into different categories of small RNAs according to their biogenesis and annotation. The most abundant RNA category from each library was miRNA.
