# Supplementary material for: Molecular Mechanism for Stress-Induced Depression Assessed by Sequencing miRNA and mRNA in Medial Prefrontal Cortex
Source: PLoS One. 2016 Jul 18;11(7):e0159093. doi: 10.1371/journal.pone.0159093 (PMC4948880; doi:10.1371/journal.pone.0159093)
Supplement: S3 Fig — (PDF) [file pone.0159093.s003.pdf]

A

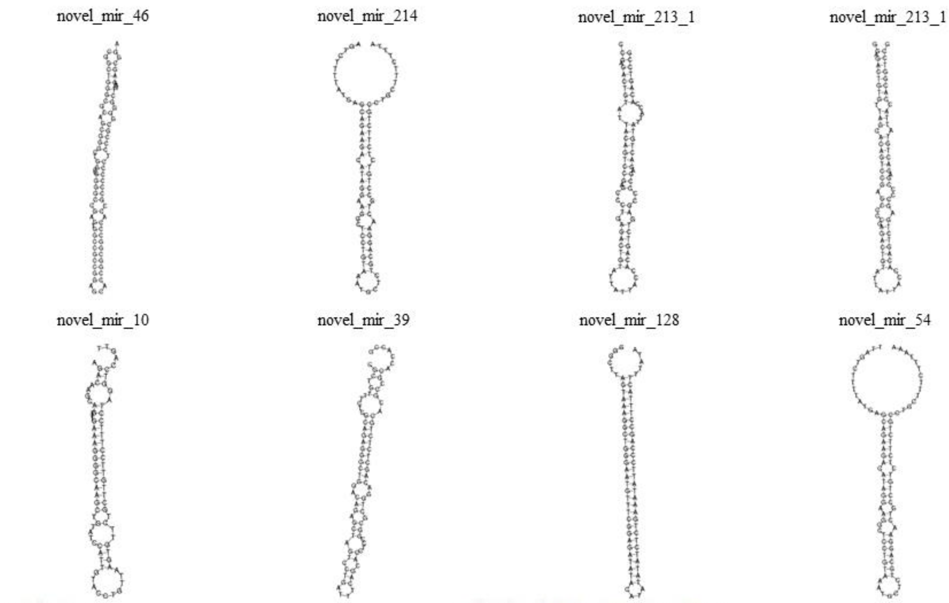

B

Novel miRNA	Predicted novel miRNA precursors sequence
novel_mir_46 ↑	CGGCTGGGCG**CAGCGGGCTGGCCGGGGCCGAGT**GGCCGCCGGAGCACCGGCGGCCCACCGCCCCCCTCCCGCGGGGCTGGACAGCGGA
novel_mir_214 ↑	AGTCTTTTATGAGCAGAAGACATAGGAAGGCTCCTGTAAATGCTCTGCAGG**AACTGCCTGTCTCTTCTGCC**TGCTTTCTTTA
novel_mir_213 ↑	CCGAGACTGTATTACAGTCCGAGCCCTGAGACTGTATTATTACCACAG**TCTGAGCCCCGAGACTGTATTA**CCACAGTCCG
novel_mir_10 ↓	AGACAAGCAG**TTTGAAAGGGGCAAGCTGTATC**CATTGTACCTGTTAAGTGTTCTGCTTGTTCCTTTCCTAGGTCCAGTT
novel_mir_39 ↓	CGCTGTTTCTCGCAGAGGGCTGGACAGAGCTAGTCCTGATTTCAGCACCTCCGG**CGCTGGACAGCTCTCTGCACGC**CGCACCACCG
novel_mir_128 ↓	GGGCTTAGTA**AAGGCTGGGAATGTTTCGGAGA**TATACATATATATCTCTGAAATATTCCCAGCCTTTACTTTATA
novel_mir_54 ↓	TTAGTCTTTTATGAGCAGAAGACATAGGAAGGCTCCTGTAAATGCTCTGCAGG**AACTGCCTGTCTCTTCTGCCTGC**TTTCTTTAAA

S3 Fig. Stem loop structure and predicted mature sequence. A: The remained reads without any annotation were used to predict potential novel miRNAs and its stem loop structure by Miredp. B: The stem loop sequence information of predict potential novel miRNAs. Red indicates the predicted novel miRNA mature nucleotide sequence.
