# Supplementary material for: Molecular Mechanism for Stress-Induced Depression Assessed by Sequencing miRNA and mRNA in Medial Prefrontal Cortex
Source: PLoS One. 2016 Jul 18;11(7):e0159093. doi: 10.1371/journal.pone.0159093 (PMC4948880; doi:10.1371/journal.pone.0159093)
Supplement: S4 Fig — (PDF) [file pone.0159093.s004.pdf]

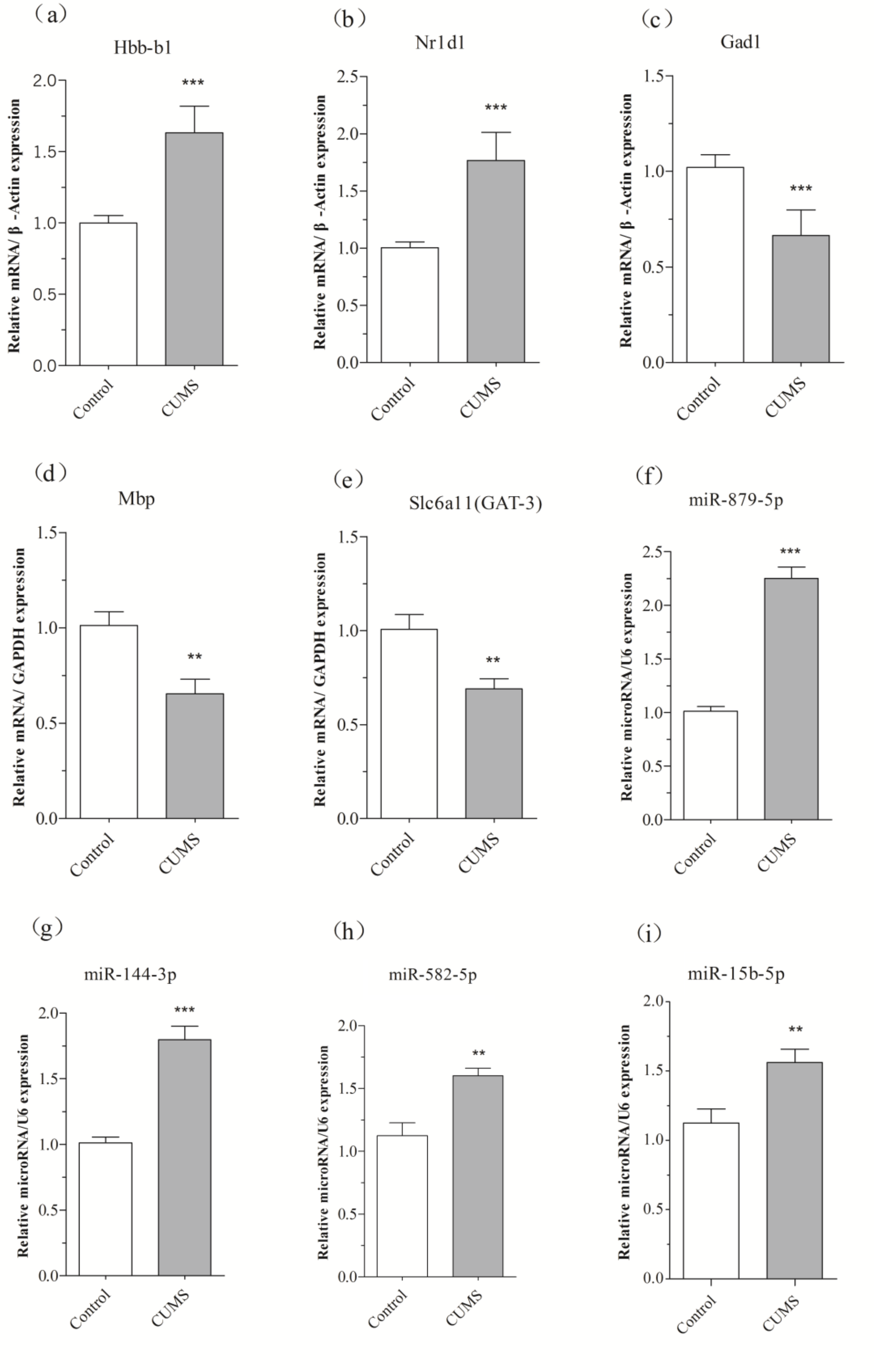

S4 Fig. The validation of differentially expressed mRNAs and miRNAs in the medial prefrontal cortex (mPFC) from CUMS-induced depression mice versus control mice.

Five mRNAs and four miRNAs that belong to different functional groups and that their expressions are significantly different between control (n=8) and CUMS-induced depression mice (n=8) were selected for qRT-PCR analysis. **a-b)** The levels of Hbb-b1 and Nr1d1 were raised in CUMS-induced depression mice. **c-e)** The levels of Gad1, Mbp and Slc6a11 were decreased in CUMS-induced depression mice. **f-i)** The four selected miRNAs (mmu-miR-879-5p, mmu-miR-144-3p, mmu-miR-582-5p and mmu-miR-15b-5p) in CUMS-induced depression mice were analyzed. The data are expressed as mean ± SEM. $^{**}p < 0.01$, $^{***}p < 0.001$.
