# Supplementary material for: Molecular Mechanism for Stress-Induced Depression Assessed by Sequencing miRNA and mRNA in Medial Prefrontal Cortex
Source: PLoS One. 2016 Jul 18;11(7):e0159093. doi: 10.1371/journal.pone.0159093 (PMC4948880; doi:10.1371/journal.pone.0159093)
Supplement: S5 Fig — (PDF) [file pone.0159093.s005.pdf]

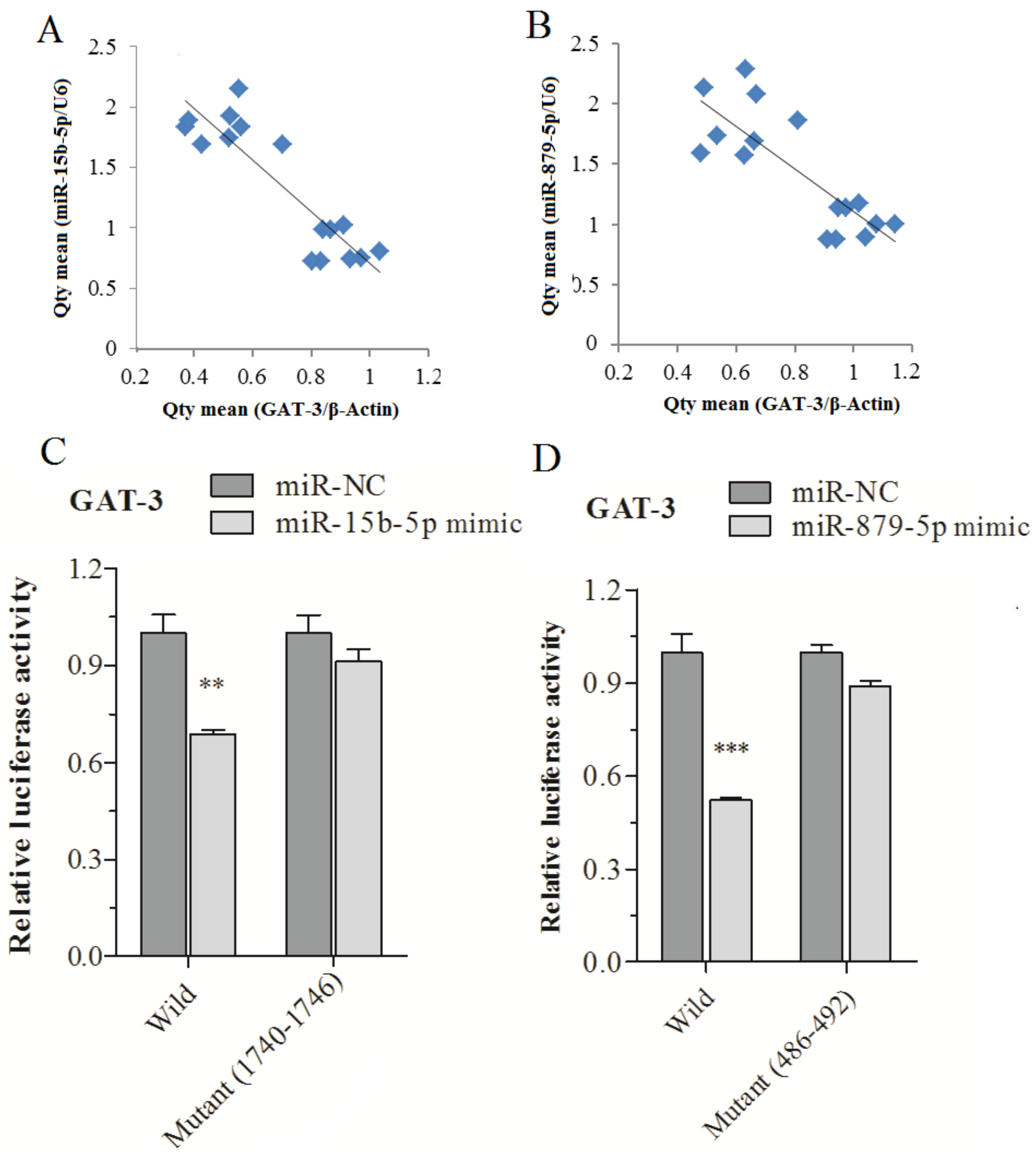

S5 Fig. The miRNAs targeted mRNAs are validated by qRT-PCR and Luciferase reporter assay. Correlation between miRNAs and its prediction target expression by qRT-PCR in PFC tissue. A) shows the correlation between GAT-3 and miR-15b-5p (r = -0.882; $p < 0.001$). B) shows the correlation between GAT-3 and miR-879-5p (r = -0.813; $p < 0.001$). C-D) Luciferase reporter assay is performed by the co-transfection of luciferase reporter containing wild or mutant 3′-UTR of GAT-3 mRNA with miRNA-15b-5p and

miRNA-879-5p mimic or their negative control (NC) into HEK293T cells. Data are the mean ± SEM. qPCR of miRNAs and mRNAs were analyzed in 8 mice per group.
