# Supplementary material for: Molecular Mechanism for Stress-Induced Depression Assessed by Sequencing miRNA and mRNA in Medial Prefrontal Cortex
Source: PLoS One. 2016 Jul 18;11(7):e0159093. doi: 10.1371/journal.pone.0159093 (PMC4948880; doi:10.1371/journal.pone.0159093)
Supplement: S1 Table — (DOCX) [file pone.0159093.s006.docx]

**S1 Table. qRT-PCR prime information**

| Gene ID | Symbol | Accession | Prime sequence | Lengths | Tm (°C） |
| --- | --- | --- | --- | --- | --- |
| 15129 | Hbb-b1 | NM_001278161.1 | Forward 5′-CCGATGAAGTTGGTGGTGA-3′ | 103 | 60 |
|  |  |  | Reverse 5′-ATAGCAGAGGCAGAGGATAGGT-3′ |  |  |
| 217166 | Nr1d1 | AK137582.1 | Forward 5′-GCAAGGCAACACCAAGAAT-3′ | 176 | 60 |
|  |  |  | Reverse 5′-GCTGAGAAAGGTCACGGAA-3′ |  |  |
| 243616 | Slc6a11 | NM_172890.3 | Forward 5′-GCCACTGGAACAACAAGGT-3′ | 149 | 60 |
|  |  |  | Reverse 5′-TCCGCAGCAGATGAAAAA-3′ |  |  |
| 17196 | Mbp | NM_001025256.2 | Forward 5′-ACCATCCAAGAAGACCCCA-3′ | 192 | 60 |
|  |  |  | Reverse 5′-ACCCCTGTCACCGCTAAAG-3′ |  |  |
| 14415 | Gad1 | AF326547.1 | Forward 5'- GGGCTATGTTCCCCTTTATGT-3' | 184 | 60 |
|  |  |  | Reverse 5'-CCTTTCTATGCCGCTGAGT-3' |  |  |
| 11461 | beta (Actb) | NM_007393.3 | Forward 5'- CTACGAGGGCTATGCTCTCC-3' | 145 | 60 |
|  |  |  | Reverse 5'- TTTGATGTCACGCACGATTT-3' |  |  |
| 2828187 | GAPDH | XM_011241214.1 | Forward 5′-CGTCCCGTAGACAAAATGGT-3′ | 110 | 60 |
|  |  |  | Reverse 5′-TTGATGGCAACAATCTCCAC-3′ |  |  |
