# Supplementary material for: Molecular Mechanism for Stress-Induced Depression Assessed by Sequencing miRNA and mRNA in Medial Prefrontal Cortex
Source: PLoS One. 2016 Jul 18;11(7):e0159093. doi: 10.1371/journal.pone.0159093 (PMC4948880; doi:10.1371/journal.pone.0159093)
Supplement: S2 Table — (DOCX) [file pone.0159093.s007.docx]

**S2 Table. Filtering transcriptome raw data and Alignment**

| Sample Name | Clean reads | Genome map rate | Gene map rate | Expressed gene | Expressed transcripts | Expressed exon | Extend gene |
| --- | --- | --- | --- | --- | --- | --- | --- |
| Ctl-1 | 45123294 | 78.84% | 72.17% | 16651 | 20372 | 189121 | 2636 |
| Ctl-2 | 45167958 | 79.41% | 73.41% | 16534 | 20228 | 188590 | 2585 |
| CUMS-1 | 45176416 | 79.62% | 74.53% | 16508 | 20183 | 186680 | 2588 |
| CUMS-2 | 45175652 | 79.58% | 72.58% | 16636 | 20335 | 188248 | 2590 |
