# Supplementary material for: Molecular Mechanism for Stress-Induced Depression Assessed by Sequencing miRNA and mRNA in Medial Prefrontal Cortex
Source: PLoS One. 2016 Jul 18;11(7):e0159093. doi: 10.1371/journal.pone.0159093 (PMC4948880; doi:10.1371/journal.pone.0159093)
Supplement: S3 Table — (DOCX) [file pone.0159093.s008.docx]

**S3 Table. Filtering small RNA library raw data and quality control**

| Control-1 |  |  | Control-2 |  |  |
| --- | --- | --- | --- | --- | --- |
| type | count | percent(%) | type | count | percent(%) |
| total_reads | 14756674 |  | total_reads | 14962143 |  |
| high_quality | 14728757 | 1 | high_quality | 14853735 | 1 |
| 3'adapter_null | 82529 | 0.0056 | 3'adapter_null | 95495 | 0.0064 |
| insert_null | 1100 | 0.0001 | insert_null | 3783 | 0.0003 |
| 5'adapter_contaminants | 11881 | 0.0008 | 5'adapter_contaminants | 31797 | 0.0021 |
| smaller_than_18nt | 145874 | 0.0099 | smaller_than_18nt | 296102 | 0.0199 |
| polyA | 4 | 0 | polyA | 10 | 0 |
| clean_reads | 14487369 | 0.9836 | clean_reads | 14426548 | 0.9712 |
| CUMS-1 |  |  | CUMS-2 |  |  |
| type | count | percent(%) | type | count | percent(%) |
| total_reads | 14297163 |  | total_reads | 14352726 |  |
| high_quality | 14285818 | 1 | high_quality | 14341362 | 1 |
| 3'adapter_null | 50744 | 0.0036 | 3'adapter_null | 33630 | 0.0023 |
| insert_null | 8221 | 0.0006 | insert_null | 2611 | 0.0002 |
| 5'adapter_contaminants | 41368 | 0.0029 | 5'adapter_contaminants | 21158 | 0.0015 |
| smaller_than_18nt | 347393 | 0.0243 | smaller_than_18nt | 236981 | 0.0165 |
| polyA | 36 | 0 | polyA | 43 | 0 |
| clean_reads | 13838056 | 0.9687 | clean_reads | 14046939 | 0.9795 |
