# Supplementary material for: Molecular Mechanism for Stress-Induced Depression Assessed by Sequencing miRNA and mRNA in Medial Prefrontal Cortex
Source: PLoS One. 2016 Jul 18;11(7):e0159093. doi: 10.1371/journal.pone.0159093 (PMC4948880; doi:10.1371/journal.pone.0159093)
Supplement: S5 Table — (DOCX) [file pone.0159093.s010.docx]

**S5 Table. miRNA changes in psychiatric disorders from other studies in comparison with our data**

| **miRNA** | **Psychiatric disease** | **Resourse** | **Modeling methods** | **Brain area** | **Assay technique** | | **Main Conclusion** | **References** |
| --- | --- | --- | --- | --- | --- | --- | --- | --- |
| miR-148b-5p ↑ | AD | Blood samples |  |  | | High-throughput sequencing | miR-148b-5p up-regulation in AD | ^1^ |
|  | SCZ | Post-mortem brain |  | PFC | | qRT-PCR | miR-148b up-regulation in SCZ | ^2^ |
|  | BD |  |  |  |  |  | miR-148b down-regulation in BD |  |
|  | ASD | Human |  | Cerebellar cortex | | Multiplex qPCR | miR-148b down-regulation in ASD | ^3^ |
|  | ASD | Human |  | Lymphoblastoid cell lines | | Microarray | miR-148b down-regulation in ASD | ^4^ |
|  | Depressed Suicide | Postmortem brain |  | PFC | | Multiplex RT-PCR plate | hsa-miR-148b down-regulation in in depressed suicide | ^5^ |
| miR-879-5p ↑ | Depression-like behavior | Mice | CUMS | Hippocampus | | Microarray | miR-879-5p up-regulation in depression model vs control | ^6^ |
| miR-144-3p ↑ | Depression-like behavior | Mice | CUMS | Hippocampus | | Microarray | miR-144-3p down-regulation depression model vs control | ^6^ |
| miR-540-5p ↑ | Depression-like behavior | Mice | CUMS | Hippocampus | | Microarray | miR-540-5p down-regulation im depression model vs control | ^6^ |
| mmiR-582-5p ↑ | Depression-like behavior | Mice | CUMS | Hippocampus | | Microarray | miR-582-5p up-regulation in depression model vs control | ^6^ |
| miR-210-5p ↑ | SCZ | Post-mortem brain |  | PFC | | qRT-PCR | miR-210 down-regulation in Schizophrenia | ^2^ |
|  | BD |  |  |  |  |  | miR-210 down-regulation in Bipolar Disorder |  |
|  | Depression-like behavior | Rat | LH | Frontal cortex | | TLDA array | miR-210 up-regulation in LH vs Controls | ^7^ |
|  | Depression-like behavior | Mice | CUMS | Hippocampus | | Microarray | miR-210-5p down-regulation in MDD vs control | ^6^ |
| miR-3103-5p ↑ | Depression-like behavior | Mice | CUMS | Hippocampus | | Microarray | miR-3103-5p up-regulation in depression model vs control | ^6^ |
| miR-15b-5p ↑ | Depression-like behavior | Rat | CUMS | Hippocampus | | Microarray | miR-15b down-regulation in depression model vs control | ^2^ |
| miR-15b-5p ↑ | SCZ | Human |  | PFC | | qRT-PCR | miR-15b down-regulation in SZ patients | ^2^ |
| miR-15b-5p ↑ | Depression-like behavior | Mice | CUMS | Hippocampus | | Microarray | miR-15b-5p up-regulation in depression model vs control | ^6^ |
| miR-15b-5p ↑ | SCZ | Human |  | Superior temporal gyrus | | Microarray, qRT-PCR | miR-15b up-regulation in SZ patients vs control | ^8^ |
| miR-16-1-3p ↑ |  |  |  |  |  |  |  |  |
| miR-16-1-3p ↑ | SCZ | Human |  | PFC | | TLDA array | miR-16 up-regulation in SZ patients vs control | ^9^ |
|  | Depression-like behavior | Rat | MD | Hippocampus | | qRT-PCR | miR-16 up-regulation in depression model vs control | ^10^ |
|  | Depression-like behavior | Mice | CUMS | Hippocampus | | Microarray | miR-16 up-regulation in depression model vs control | ^6^ |
| let-7a-1-3p ↑ | Depression-like behavior | Mice | immobilization stress | Hippocampal CA1 | | Spotted array | Whether under acute or chronic stress mmu-let-7a-1 was down | ^11^ |
|  |  | Rat | MD and CUMS | Hippocampus | | qRT-PCR | Higher Let-7a level in the hippocampus than control rats | ^10^ |
|  |  | Mice | CUMS | Hippocampus | | Microarray | Llet-7a up-regulation in depression model vs control | ^6^ |
| miR-470-5p ↑ | Depression-like behavior | Mice | CUMS | Hippocampus | | Microarray | miR-470-5p up-regulation in depression model vs control | ^6^ |
| miR-218-2-3p ↑ | Depression-like behavior | Mice | CUMS | Hippocampus | | Microarray | miR-218-2-3p up-regulation in depression model vs control | ^6^ |
| miR-190-5p ↑ | BD | Human |  | PFC | | qRT-PCR | miR-190b-5p down-regulation in BD patients | ^2^ |
|  | Depressed Suicide | Postmortem brain |  | PFC | | Multiplex RT-PCR plate | hsa-miR-190 down-regulation in depressed suicide | ^5^ |
|  |  |  |  |  | |  |  |  |

Abbreviations: ↑ indicates miRNA is up-regulation in the tissue of PFC from depression-like mice versus control mice, whereas ↓ represent down-regulation. AD, Alzheimer’s disease; SCZ, Schizophrenia; BD, Bipolar Disorder; ASD, Autism Spectrum Disorders; CUMS, Chronic unpredicted mild stress; MD, Maternal deprivation; LH, Learned helplessness, PFC, Prefrontal cortex
